# Reactive oxygen species: balancing agents in plants

**DOI:** 10.3389/fpls.2025.1713590

**Published:** 2025-12-08

**Authors:** Pravej Alam, Mohammad Faizan, Yamshi Arif, Maged M. Azzam, Shamsul Hayat, Shadma Afzal, Thamer Albalawi

**Affiliations:** 1 Department of Biology, College of Science and Humanities, Prince Sattam Bin Abdulaziz University, Alkharj, Saudi Arabia; 2 Botany Section, School of Sciences, Maulana Azad National Urdu University, Hyderabad, India; 3 Department of Botany, Faculty of Life Sciences, Aligarh Muslim University, Aligarh, India; 4 Department of Chemistry, College of Science and Humanities, Prince Sattam Bin Abdulaziz University, Al-Kharj, Saudi Arabia; 5 Department of Bioclimatology, Faculty of Environmental Engineering and Mechanical Engineering, Poznan University of Life Sciences, Poznań, Poland

**Keywords:** antioxidant, signaling, phytohormones, proteins, homeostasis, stress

## Abstract

Reactive oxygen species (ROS) are highly reactive molecules derived from molecular oxygen, playing a dual role in plant systems as both signaling molecules and potential agents of cellular damage. This comprehensive review highlights the fundamental aspects of ROS biology in plants, beginning with the definition and chemical nature of ROS, followed by an in-depth discussion of their various types, including singlet oxygen (1O_2_), superoxide radicals (O_2_•^−^), hydrogen peroxide (H_2_O_2_), and hydroxyl radicals (•OH). The review outlines the primary sites of ROS production within plant cells, such as mitochondria, chloroplasts, and peroxisomes, and explains their integral roles throughout the plant life cycle, encompassing growth, development, and senescence. Furthermore, the involvement of ROS in cell-cycle regulation, cell division, and programmed cell death is discussed, emphasizing their critical role in maintaining cellular homeostasis. The review also sheds light on ROS-mediated signaling pathways and their interactions with key plant hormones, including strigolactones, salicylic acid, brassinosteroids, jasmonic acid, and karrikins, highlighting the complexity of ROS-hormone cross talk in regulating stress responses and development. The damaging effects of uncontrolled ROS accumulation on DNA, lipids, proteins, and enzymes are thoroughly examined, underscoring their potential to disrupt cellular functions. Methods for detecting ROS in plant tissues are briefly presented, offering insights into current techniques used for quantifying and visualizing ROS. Overall, this review provides a detailed understanding of ROS dynamics in plant biology and serves as a valuable reference for future research aimed at manipulating ROS signaling to enhance plant resilience and productivity.

## 1 What is ROS?

Reactive oxygen species (ROS) are a group of highly reactive free radicals and molecules derived from molecular oxygen (O_2_) (Nafees et al., 2019). In plants, ROS are mainly produced as by-products of cellular metabolism (de Almeida et al., 2022). Initially, ROS were considered merely toxic by-products of aerobic metabolism. However, recent research has demonstrated that ROS also play crucial signaling roles in regulating plant growth, development, and responses to various biotic and abiotic stresses (Das and Roychoudhury, 2014). Despite their destructive potential, ROS are now recognized as important second messengers involved in a wide range of cellular processes, including the induction of tolerance mechanisms under environmental stress conditions (Faraz et al., 2023; Alyemeni et al., 2016). The delicate balance between ROS generation and scavenging determines whether ROS act as signaling molecules or cause oxidative damage to cellular components (Shareen et al., 2023). Effective ROS detoxification during stress conditions relies on the coordinated activity of enzymatic and non-enzymatic antioxidant defense systems in plant tissues. The major members of the ROS family include free radicals such as O_2_•^−^ and •OH, as well as non-radical molecules like H_2_O_2_ and 1O_2_.

The objective of this review is to comprehensively elucidate the biological significance of ROS in plants by examining their origin, types, and primary sites of production. It aims to explore the dual role of ROS as signaling molecules and as agents of oxidative damage, along with their involvement in plant growth, development, and stress responses. Furthermore, the review seeks to highlight the intricate cross talk between ROS and plant hormones and to discuss current methods used for ROS detection. Ultimately, this work aspires to provide insights that may facilitate the manipulation of ROS signaling for improved plant resilience and productivity.

## 2 Chemistry of ROS

The high reactivity of oxygen is attributed to the presence of two unpaired electrons in its outer orbital, which influence its chemical behavior and ability to form ROS. In plant cells, mitochondria and chloroplasts are the two primary sites of ROS production. During photosynthesis, chlorophyll pigments in the chloroplast absorb light energy and become excited to a triplet state. This excited state is short-lived (approximately 3.1–3.9 µs) and can transfer energy to molecular oxygen, leading to the formation of 1O_2_ (Meriga et al., 2004). Similarly, molecular oxygen released at the terminal step of the mitochondrial electron transport chain may undergo partial reduction, generating ROS such as O_2_•^−^ and H_2_O_2_. Not all ROS are free radicals; non-radical species such as H_2_O_2_ and ozone (O_3_) are also common in plants. Other reactive oxygen-related molecules include carbonyl compounds, hypochlorous acid (HOCl), hypobromous acid (HOBr), and carbonate radicals (CO_3_•^−^). Collectively, these ROS species are formed as by-products of incomplete oxygen reduction processes occurring during normal metabolic activities or under stress conditions (Hussain et al., 2023).

Whenever a plant is exposed to abiotic stress, it experiences oxidative stress that leads to the excessive generation of ROS. The redox potential and enzymatic antioxidant activities work in coordination to maintain cellular equilibrium under such stress conditions. Several antioxidant enzymes in plant cells actively participate in sustaining cellular metabolism during stress by regulating ROS levels. As mentioned earlier, a critical threshold of ROS must be maintained for normal metabolic and physiological functions. Both ROS production and scavenging are tightly regulated by specific signaling proteins within the cell (Hongyu et al., 2023; Huizhi et al., 2022). Consequently, ROS are recognized not only as harmful by-products but also as essential signaling molecules that modulate various physiological and metabolic processes (Evans et al., 2004; Mohiuddin et al., 2023). However, excessive accumulation of ROS under stress conditions disrupts this balance, leading to oxidative damage to cellular components. Such imbalance between ROS generation and scavenging disturbs metabolic activities and impairs photosynthetic efficiency (Bilge et al., 2025; Gulsah et al., 2025). This oxidative damage manifests as chlorophyll photo-bleaching, enhanced lipid peroxidation, reduced protein content, and increased membrane injury (Kumari et al., 2025). To counteract these effects, several antioxidant enzymes such as catalase (CAT), superoxide dismutase (SOD), and peroxidases (POD, including lipid peroxidase) are activated to scavenge ROS and protect the cell from stress-induced injury (Alam et al., 2025).

## 3 Types of ROS

ROS have become integral by-products of aerobic life since the evolution of molecular O_2_ in the atmosphere, primarily produced by oxygenic photosynthetic microorganisms. In plants, ROS are continuously produced under normal physiological conditions within membrane-bound organelles such as mitochondria, chloroplasts, and peroxisomes, as well as in other cellular compartments. Their formation is closely associated with key metabolic pathways, including photosynthesis, photorespiration, and respiration.

### 3.1 Singlet oxygen (^1^O_2_)

Singlet oxygen, or commonly known as a triplet oxygen, is considered to be the first excited electronic state of molecular oxygen which is produced in plant leaves in sunlight by the reaction chlorophyll triplet state with O_2_ in the chloroplast antenna system.


$$\text{Chl}\to^{3}\text{Chl}$$

$$3\text{Chl}{+}^{3}{\text{O}}_{2}\to \text{Chl}{+}^{1}{\text{O}}_{2}$$

^1^O_2_ has a short lifespan, which suggest a small diffusion route in plant cells. Depending on environmental conditions, singlet oxygen can persist for 1 h at room temperature, due to its exceptional properties. Singlet and triplet oxygen has different chemical properties due to the differences in their electron shells. The major source for the generation of singlet oxygen is the chlorophyll pigments which are associated with the electron transport process. During photosynthesis, insufficient energy disintegration can cause the chlorophyll triplet state formation which can transfer its excitation energy onto the ground state O_2_ to make ^1^O_2_. On the contrary, ^1^O_2_ can also be produced as a derivative of lipoxygenase activity. Like other ROS, ^1^O_2_ is extremely catastrophic in nature as it can react with most of the biomolecules and, hence, can be fatal to plant cells and can activate two types of responses involving signaling of acclimation processes or programmed cell death. In the electron transport chain of photosynthesis, the transfer of energy or electrons takes place by the excited singlet state of chlorophyll (Ahmad et al., 2025; Yongchao et al., 2023). Moreover, ^1^O_2_ has the ability to diffuse from the chloroplasts into the apoplast and the cytoplasm. Exposures to environmental stresses increases levels of ^1^O_2_ in root cells of the plants. Recent studies of photo-oxidative damage to plant tissues, in association with the lipid peroxidation processes, reported that the main ROS involved in the leaves destruction is ^1^O_2_.

### 3.2 Superoxide radical (O_2_^•–^)

Superoxide radical is an oxygen compound produced, and the primary form of ROS produced by the mitochondria exists commonly in nature. The formation of superoxide radical takes place in the photosystem I (PSI) of the thylakoid membrane, produced by the non-cyclic electron transport chain. The factors that determine the redox state of the ferredoxin pool includes the controlled activation of Calvin cycle and regulated electron flow rate. According to Asada and Takahashi (1987), it is important for the electron carriers and the ferredoxins present on the reduced side of PSI, possessing negative electrochemical potentials necessary for donating electrons to oxygen atoms, thus leading to the superoxide radical formation.


$${\text{O}}_{2}^{•–}+{\text{Fe}}^{3+}\to^{1}{\text{O}}_{2+}{\text{Fe}}^{2+}$$

$${\text{O}}_{2}^{•–}+2{\text{H}}^{+}\to {\text{O}}_{2}+{\text{H}}_{2}{\text{O}}_{2}{\text{Fe}}^{3+}$$

$${\text{Fe}}^{2+}+{\text{H}}_{2}{\text{O}}_{2}+{\text{Fe}}^{3+}\to {\text{OH}}^{-}+{\text{OH}}^{•}$$

O_2_^•–^ have a short half-life of 2-4 μs and is highly reactive in nature. It changes to hydroxy radicals, which results in membrane dissolution and immense lipid peroxidation (Halliwell, 2006).

### 3.3 Hydrogen peroxide (H_2_O_2_)

Hydrogen peroxide is a compound with an oxygen–oxygen single bond. H_2_O_2_ is slightly a ROS as well as the simplest form of peroxide. H_2_O_2_ is a clear liquid that is somewhat more viscous than water and is colorless in dilute solution. It is formed through monovalent reduction and protonation of O_2_^•–^. Superoxide radical generation is caused by the reaction catalyzed by the superoxide dismutase (SOD) activity:


$$2{\text{O}}_{2}^{•–}+2{\text{H}}^{+}\to {\text{H}}_{2}{\text{O}}_{2}+{\text{O}}_{2}$$

**Figure 1:**
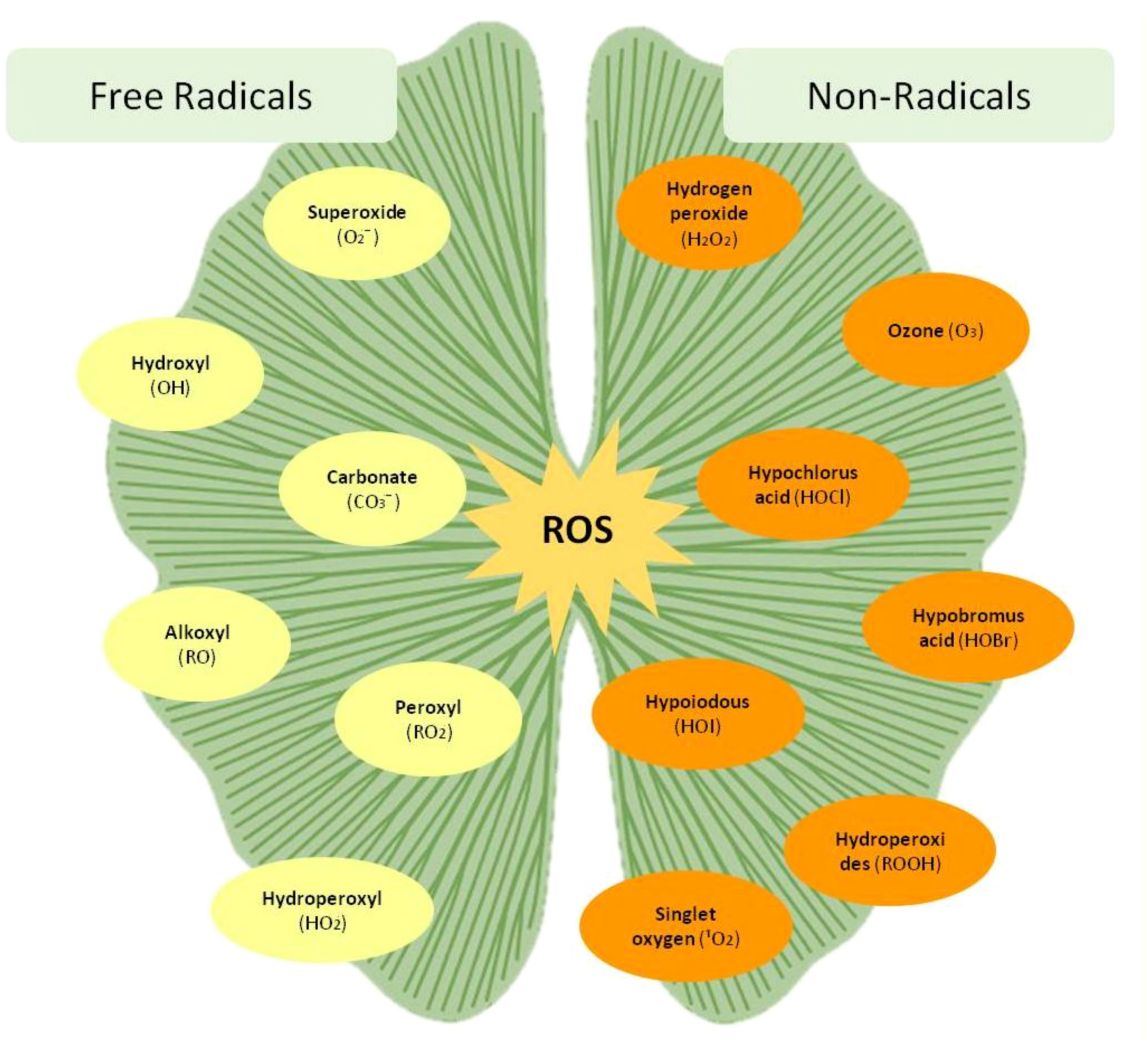
Classification of ROS into free radicals and non-radicals in plants.

In plant cells, multiple sources caused the production of H_2_O_2_ such as the electron transport chain (ETC) in mitochondria or chloroplasts, cell membrane, endoplasmic reticulum (ER), photo-respiration, and lipid peroxidation. It is also produced in response to the oxidative stress caused by several environmental factors like UV radiation, high light intensity, salinity, drought, and pathogen attack. H_2_O_2_ like every other ROS acts as a double-edged sword; it is favorable at lower concentrations, but at the same time can be toxic at higher concentrations in the plant cells. It also acts as a key regulator for several important physiological processes like photosynthesis and photorespiration, senescence (Zhixin et al., 2022), stomatal movement, cell cycle, and plant growth and development. Compared with other ROS members, it has a half-life of 1 ms, due to which it can travel longer distances and can traverse plant cell membranes (**Figure 1**).

### 3.4 Hydroxyl radical (OH•)

Among all the ROS members, hydroxyl radical (OH^•^) is the neutral form of the hydroxide ion, highly reactive due to its high reduction potential, and the most carcinogenic ROS known till now. OH^•^ has a very short half-life of 10^−9^ s. OH^•^ is generated by the Fenton reaction between H_2_O_2_ and O_2_^•–^ catalyzed by the Fe^2+^ or Fe^3+^ at neutral pH.


$${\text{O}}_{2}^{•-}+{\text{H}}^{+}+{\text{H}}_{2}{\text{O}}_{2}\to {\text{O}}_{2}+{\text{OH}}^{•}+{\text{H}}_{2}\text{O}$$

Moreover, the initial formation of O_2_ occurs when O_2_^•−^ undergoes stepwise monovalent reduction. This O_2_^•−^ is formed and then acts as an electron donor in the hydroxyl radical production by the Haber–Weiss reaction. The Haber–Weiss reaction has a finite role in the generation of superoxide and OH^•^, formulated as the H_2_O_2_ source which causes hydroxyl radical production via the Fenton reaction. OH^•^ has the great potential of damaging different cellular components by cell membrane destruction, protein damage, and β-oxidation of fatty acids. In addition, increase in the concentration of OH^•^ in the plant cells causes cellular death.

## 4 Site of ROS production in plants

**Figure 2:**
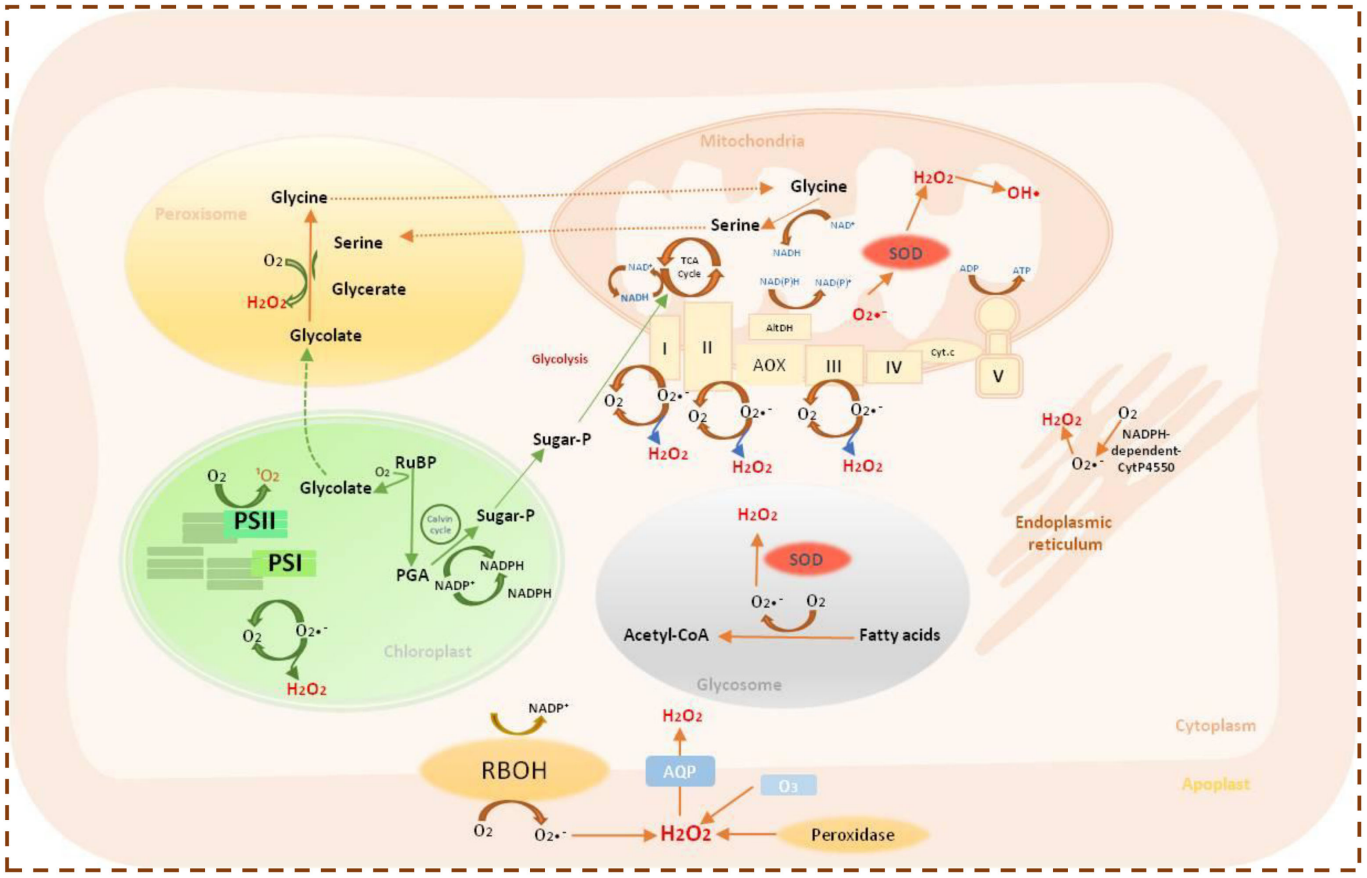
Schematic representation of ROS generation and metabolism in different subcellular compartments of plant cells.

In plants, various environmental stresses such as extreme temperatures, high light intensity, salinity, drought, heavy metals, air pollutants, pests, and pathogen attacks lead to excessive production of ROS. Several cellular organelles, including mitochondria (Huang et al., 2016), chloroplasts (Dietz et al., 2016), and peroxisomes (Sandalio and Romero-Puertas, 2015), serve as major sites of ROS generation. Approximately 1% of the oxygen consumed by plants is converted into ROS within subcellular compartments such as peroxisomes, chloroplasts, and mitochondria (Sandalio and Romero-Puertas, 2015; Huang et al., 2016). ROS generation occurs primarily through electron transport systems, as molecular O_2_ acts as a strong terminal electron acceptor. Molecular O_2_ undergoes stepwise reduction, yielding several reactive intermediates. In the first step, the one-electron reduction of O_2_ produces O_2_•^−^, which has a short half-life of 2–4 µs and limited diffusion capacity (Halliwell, 2006). Subsequent reduction leads to the formation of H_2_O_2_, a relatively stable molecule with a lifetime of approximately 1 ms. Hydrogen peroxide can form spontaneously through dismutation of O_2_•^−^ under acidic conditions or more efficiently in the presence of the enzyme SOD (Das and Roychoudhury, 2014). In the presence of transition metal catalysts, •OH are produced via Haber–Weiss or Fenton-type reactions. The hydroxyl radical is extremely reactive, attacking nearly all biomolecules near its site of formation and causing severe oxidative damage. The major cellular sites and pathways responsible for ROS production are discussed in this section, and an overview is presented in **Figure 2**.

### 4.1 Mitochondria

Multiple stresses can generate ROS in mitochondria by hindering and modifying the ETC and ATP generation, resulting in a surplus reduction in electron carriers and the generation of ROS (Noctor et al., 2007). Electron leakage from complexes I and III of the ETC generates O_2_•^−^, which is catalyzed into H_2_O_2_ by Mn-SOD and CuZn-SOD. The flavoprotein region of complex I is responsible for the direct reduction of O_2_ to O_2_•^−^. Reverse electron transport from complex III to complex I as a result of an insufficiency of NAD^+^-linked substrate enhances ROS generation at complex I, and this reverse electron movement is controlled by ATP hydrolysis (Turrens, 2003). The ETC has a ubiquinone-cytochrome region (complex III) which also generates O_2_•^−^ from O_2_. Completely reduced ubiquinone is thought to donate an electron to cytochrome c_1_ and leave behind an unstable, extremely reducing ubisemiquinone radical that is conducive to electron leakage to O_2_, subsequently leading to the generation of O_2_^•−^ (Murphy, 2009). Moreover, mitochondrial matrix enzymes that contribute to ROS production include aconitase, which produces ROS directly, and 1-galacto-lactone dehydrogenase, which contributes to ROS production indirectly by providing electrons to the ETC (Rasmusson et al., 2008). O_2_•^−^ is the predominant ROS in mitochondria. However, Mn-SOD and ascorbate peroxidase (APX) quickly convert it into stable and membrane-permeable H_2_O_2_, which undergoes further conversion to the extremely reactive ^•^OH through the Fenton reaction.

### 4.2 Chloroplast

In chloroplast, triplet chlorophyll (^3^Chl), ETC of photosystem I (PSI), and photosystem II (PSII) are the primary sources of ROS generation (Singh et al., 2019). Under the influence of light, the chlorophyll in the PSII light-harvesting complex reaches a high-energy singlet state after becoming excited. By photochemical quenching, a portion of this energy is transferred to P680 to power the photosynthetic ETC. However, when the amount of energy absorbed surpasses the capacity of the photochemical quenching, the extra energy is released as heat, as fluorescence, or through the intersystem crossing, forming triplet excited chlorophyll (^3^Chl*). If ineffective quenching of this triplet excited chlorophyll (^3^Chl*) occurs, it will undergo a redox reaction with ^3^O_2_ liberated during the splitting of water in the oxygen-evolving complex (OEC), producing ^1^O_2_ (Li et al., 2009). Furthermore, P680 is excited to the singlet state (^1^P680*) after absorbing light in the PSII Reaction Center (RC), after which it couples with pheophytin (Pheo) to form ^1^(P680^+^ Pheo^-^) and finally transfers an electron to the quinone (Q_A_) to form P680^+^ Q_A_^−^. In an adverse circumstance, if Q_A_ has been reduced to the point where it cannot accept any additional electrons, the excited state ^3^P680* is formed when ^3^(P680^+^ Pheo^−^) recombines with P680 (Krieger-Liszkay, 2005). Two molecules of β-carotene are present in the PS II RC and can quench this high-energy ^3^P680*; since they are too far apart, quenching is unable to take place, which results in the production of ^1^O_2_ (Krieger-Liszkay et al., 2008). Additionally, in PS II, stomatal closure due to the abiotic stresses minimizes the chloroplastic CO_2_ concentration, leading to the overproduction of the ETC and increasing the chance of electronic conductivity between ^1^P680* and Q_A_, resulting in an increase in ^1^O_2_ generation. On the other hand, O_2_^•−^ can be formed through the Mehler reaction and then transformed into H_2_O_2_ by SOD, as opposed to ^1^O_2_ being made at PS II (Bose et al., 2014). Subsequently, metal catalysts like Fe^2+^ transform O_2_^•−^ and H_2_O_2_ into the much more unstable and reactive ^•^OH (Singh et al., 2019).

### 4.3 Peroxisomes

Similar to mitochondria and chloroplasts, peroxisomes also generate ROS as by-products of normal cellular metabolism. The enzyme glycolate oxidase (GOX) is the principal source of ROS within peroxisomes, where it catalyzes the oxidation of glycolate to glyoxylate, producing H_2_O_2_ as a by-product (Kerchev et al., 2016). In addition, xanthine oxidase located in the peroxisomal matrix converts xanthine and hypoxanthine to uric acid, generating O_2_•^−^ as secondary products. The peroxisomal membrane also harbors a small NAD(P)H-dependent ETC composed of NADH and cytochrome b, which uses molecular O_2_ as the terminal electron acceptor, releasing O_2_•^−^ into the cytosol. Furthermore, three integral peroxisomal membrane polypeptides (PMPs) with molecular masses of approximately 18, 29, and 32 kDa have been identified as key contributors to O_2_•^−^ generation. The 18- and 32-kDa PMPs utilize NADH as the electron donor to reduce cytochrome c, whereas the 29-kDa PMP employs NADPH for the same function.

## 5 ROS in life cycle of plant

ROS are unavoidable byproducts of cellular metabolism in nearly all plant cells. While they were once regarded primarily as indicators of oxidative stress, recent research has established their essential signaling roles in diverse physiological and developmental processes throughout the plant life cycle (Mhamdi and Van Breusegem, 2018). These include seed development and germination, as well as the growth and differentiation of roots, shoots, and flowers, emphasizing the dual role of ROS as both stress mediators and key regulators of plant growth and development (Singh et al., 2016). Seed dormancy and germination are tightly regulated physiological processes controlled by intricate networks of signaling pathways. Germination begins with water uptake (imbibition), which activates metabolism, cell division, and ultimately radicle protrusion (Bewley and Black, 2013). In dry, dormant seeds, metabolic activity in the embryo and endosperm is minimal, leading to very low ROS production (Singh et al., 2016). Upon imbibition, however, rapid metabolic reactivation occurs, accompanied by a marked increase in ROS generation through multiple pathways and subcellular sites, including NADPH oxidases, lipid catabolism, β-oxidation in glyoxysomes, and mitochondrial respiration (Ishibashi et al., 2017). During this phase, metabolically active organelles such as chloroplasts (via photosystems), glyoxysomes (through lipid metabolism), mitochondria (via the electron transport chain), peroxisomes (during purine catabolism), and plasma membranes (via NADPH oxidase) become major contributors to ROS formation (Singh et al., 2016). Among these, mitochondria play a central role, producing H_2_O_2_ as a result of electron leakage from the respiratory chain. Unlike dry seeds where ROS activity remains localized, hydrated seeds permit the diffusion of ROS, particularly the more stable H_2_O_2_, allowing them to act at distant cellular targets (Bailly et al., 2008). During seed imbibition, ROS levels increase within a specific “oxidative window” that promotes germination. Both insufficient and excessive ROS levels can impair this process and too little fails to trigger signaling events necessary for germination, whereas excessive accumulation damages cellular structures and reduces embryo viability (Singh et al., 2016). Hence, maintaining ROS homeostasis is crucial for proper seedling establishment. ROS also interact intricately with phytohormones, particularly gibberellic acid (GA) and abscisic acid (ABA), to regulate germination and early growth. In cereal grains, ROS mediate programmed cell death (PCD) in the aleurone layer through cross talk with GA and ABA (Miransari and Smith, 2014; Corbineau et al., 2014). GA promotes germination and post-germinative events by downregulating ROS-scavenging enzymes, whereas ABA maintains dormancy by sustaining their activity. Additionally, ROS facilitate endosperm weakening, cell wall loosening, and radicle elongation by modifying cell wall polysaccharides and activating Ca^2+^ signaling (Muller et al., 2009). H_2_O_2_ also enhances GA-induced α-amylase synthesis in aleurone cells, whereas antioxidants such as CAT, APX, and SOD suppress it (Nonogaki, 2014). Conversely, ABA antagonizes GA by activating opposing gene networks, illustrating the delicate hormonal balance regulated by ROS. Experimental evidence further supports the stimulatory role of ROS in germination. H_2_O_2_ enhances germination and alleviates ABA-induced inhibition in Arabidopsis and pea seeds through GA-dependent pathways (Singh et al., 2016). In barley, H_2_O_2_ relieves dormancy primarily by enhancing GA synthesis and signaling rather than repressing ABA activity (Bahin et al., 2011). Collectively, these studies underscore that ROS act as key modulators of seed germination by mediating the hormonal interplay between GA and ABA, ensuring the proper transition from dormancy to seedling establishment.

Following germination, seedlings perceive gravity and exhibit gravitropism growth, directing roots downward (positive gravitropism) and shoots upward (negative gravitropism). This coordinated response relies on the asymmetric redistribution of auxin, leading to differential growth and organ bending. Gravitropic signaling has been closely linked to ROS generation in the roots of Arabidopsis and maize (Joo et al., 2005). In gravistimulated roots, ROS initially accumulate asymmetrically in the lower cortex and later become symmetrically distributed during prolonged stimulation (Singh et al., 2016). Exogenous H_2_O_2_ application can induce root bending, suggesting its role in modulating cell wall extensibility. Moreover, ROS scavengers such as N-acetylcysteine (NAC) inhibit curvature without affecting growth, indicating that asymmetric ROS production drives gravitropic curvature by locally restricting root elongation on the lower flank (Singh et al., 2016). In both the shoot apical meristem (SAM) and root apical meristem (RAM), stem cells are organized around a central zone (CZ) and an organizing center OZ in shoots and the quiescent center (QC) in roots. Their maintenance depends on intricate signaling between these zones and feedback from surrounding differentiated tissues. SAM activity is primarily governed by the WUSCHEL (WUS)–CLAVATA (CLV) regulatory loop, whereas RAM maintenance involves SCARECROW (SCR), SHORT ROOT (SHR), and PLETHORA (PLT) transcription factors (Mhamdi and Van Breusegem, 2018). Both meristems are influenced by interactions between ROS, redox components, and phytohormones (Schippers et al., 2016). The RAM is particularly sensitive to changes in cellular redox status exposure to H_2_O_2_ which reduces the number of meristematic cells, indicating its inhibitory effect on meristem activity (Tsukagoshi et al., 2010). Similarly, DNA damage induces H_2_O_2_ accumulation via FLAVIN-CONTAINING MONOOXYGENASE 1 (FMO1), leading to decreased meristem size, further supporting the role of H_2_O_2_ as a negative regulator of RAM activity (Tsukagoshi et al., 2010; Chen and Umeda, 2015). Distinct ROS gradients exist across root zones, with O_2_•– peaking in the cell division zone and H_2_O_2_ in the elongation zone, revealing their antagonistic functions in root growth (Tsukagoshi, 2016). In the SAM, a similar redox-based antagonism is observed; superoxide enhances WUS transcription, maintaining stem cell identity, whereas H_2_O_2_ inhibits this activity and promotes cell differentiation in the peripheral zone (Zeng et al., 2017; Mhamdi and Van Breusegem, 2018). The final size and morphology of plant organs result from the finely tuned coordination between cell proliferation and cell expansion (Lu et al., 2014). During leaf development, an initial phase of active cell division transitions to a subsequent phase dominated by cell expansion, during which further cell divisions cease (Beemster et al., 2005). This transition is regulated by a complex interplay of transcription factors that act as positive or negative regulators of growth (Townsley and Sinha, 2012). Cell expansion is largely influenced by modifications in cell wall architecture and composition (Singh et al., 2016). Apoplastic peroxidases (Prxs) play a crucial role in modulating cell wall stiffness by influencing ROS levels; superoxide promotes wall loosening and expansion, whereas H_2_O_2_ induces rigid cross-linking, thereby restricting growth (Tsukagoshi et al., 2010; Lu et al., 2014).

The MYB-like transcription factor KUA1 in Arabidopsis thaliana regulates apoplastic ROS homeostasis to promote cell expansion. Overexpression of KUA1 results in larger cells and leaves, whereas kua1–1 mutants display smaller leaves due to reduced cell size, elevated H_2_O_2_ accumulation, and enhanced Prx activity. Thus, KUA1-mediated ROS regulation is essential for optimal cell expansion and organ size determination (Lu et al., 2014). Apoplastic H_2_O_2_ levels play a central role in controlling cell expansion, with H_2_O_2_ also influencing the O_2_•– pool (Singh et al., 2016). Suppression of Prx expression by KUA1 enhances leaf cell expansion without affecting cell proliferation (Lu et al., 2014). Conversely, exogenous H_2_O_2_ treatment reduces root cortical cell size, indicating that root-localized Prxs maintain low H_2_O_2_ levels to sustain expansion (Tsukagoshi et al., 2010). Inhibition of Prx activity enhances leaf growth, demonstrating that apoplastic Prxs generate H_2_O_2_ that promotes cell wall cross-linking and restricts expansion (Lu et al., 2014). Collectively, these findings reveal the dual and opposing effects of Prxs on plant growth, driven by their regulation of H_2_O_2_ levels. ROS also play vital roles in the development of reproductive organs and tissues. The glutathione/glutaredoxin (GRX) redox system, particularly class III CC-type GRXs known as ROXYs, has been implicated in flower development (Gutsche et al., 2015). In Arabidopsis, roxy1 mutants exhibit defective petal formation, whereas PETAL LOSS (ptl) mutant phenotypes are influenced by ROXY1 function. PTL and ROXY1 cooperate to regulate sepal and petal initiation and inter-organ growth (Lampugnani et al., 2013; Quon et al., 2017). ROXY1 functions through interactions with TGA transcription factors, such as PERIANTHIA and TGA2/3/7, to coordinate floral organ development (Mhamdi and Van Breusegem, 2018).

ROS accumulation at the pollen tube tip is essential for its directed growth toward the female gametophyte (Potocký et al., 2012). Calcium-dependent activation and phosphorylation of NADPH oxidases (RBOHH and RBOHJ) regulate tip-localized ROS production; mutations in these genes cause growth oscillations and tube collapse (Duan et al., 2014; Lassig et al., 2014). ROP1-mediated localization of NADPH oxidases orchestrates ROS production and guides pollen tube elongation (Kaya et al., 2014). ROS also influence cell wall extensibility, facilitating pollen tube penetration into female tissues (Smirnova et al., 2014). Loss-of-function mutations in RBOHE or RBOHC lead to pollen abortion and reduced fertility (Xie et al., 2014). In female gametophytes, mitochondrial ROS—particularly those produced by manganese superoxide dismutase (MSD1)—are required for proper embryo sac development (Martin et al., 2014). During pollen tube expansion, a tip-focused cytoplasmic _3_ (cyt_3_) gradient and apoplastic ROS (apoROS) production are tightly coordinated. _3_ is supplied from the apoplast or cell wall, with plasma membrane H^+^-ATPases (AHAs) regulating its release (Singh et al., 2016). Intracellular _3_ stored in vacuoles and the ER-Golgi system is mobilized to maintain cyt_3_ homeostasis via P-type IIB _3_-ATPases (ACAs) and CAX antiporters. Elevated cyt_3_ activates NADPH oxidases, enhancing ROS generation, which in turn stimulates further _3_ influx, forming a positive feedback loop (Singh et al., 2016). RBOHH/J and RBOHC link ROS production to _3_-activated signaling pathways in pollen tubes. A localized ROS burst is also necessary for pollen tube rupture and sperm release (Duan et al., 2014). Oscillatory pollen tube growth is regulated by pH changes mediated by cation (H^+^)/anion (OH^−^) channels, H^+^-ATPases, and _3_/H^+^ exchangers. Although the exact AHA isoform responsible for apoplastic proton export in pollen tubes remains unknown, AHA2 is strongly expressed in growing root hairs, suggesting a similar role (Singh et al., 2016). Apoplastic pH directly affects enzymatic processes that remodel the cell wall during expansion (Altartouri and Geitmann, 2015). Coordinated oscillations in _3_, ROS, and pH transiently loosen the cell wall, enabling turgor-driven localized growth (Spartz et al., 2014; Wolf and Höfte, 2014).

## 6 ROS in cell cycle, cell division, and cell death

In plants, exposure to stress often results in reduced growth and cell cycle arrest, although the underlying mechanisms remain only partially understood. Redox cycles are known to be conserved throughout the cell cycle, where both oxidative and reductive signals play essential roles in phase transitions (Diaz-Vivancos et al., 2013; De Simone et al., 2017). These transitions between different cell cycle phases are primarily regulated by a complex network of cyclins (CYCs) and cyclin-dependent kinases (CDKs). Recent studies have elucidated how ROS and redox fluctuations modulate the expression and activity of these key regulatory proteins (Foyer et al., 2018). Redox reactions directly influence cell cycle components through TEOSINTE BRANCHED1-CYCLOIDEA-PROLIFERATING CELL FACTOR1 (TCP) transcription factors, which regulate the transcription of CYCs by interacting with their promoters (Kadota et al., 2005). TCPs contain a conserved redox-sensitive cysteine residue crucial for DNA binding; under oxidizing conditions, disulfide bond formation may inhibit TCP-promoter interactions, thus modulating cell cycle progression (Viola et al., 2016). Glutathione, the primary redox buffer in plant cells, is indispensable for maintaining redox equilibrium during cell division, as evidenced by growth defects in rml1 mutants deficient in glutathione biosynthesis (García-Giménez et al., 2013).

A dynamic redox cycle involving oscillations in ROS, ascorbate, and glutathione levels regulates progression through specific cell cycle checkpoints (Tognetti et al., 2017). Proper control of ROS levels is also crucial for cytokinesis. Disruption of ROS homeostasis either pharmacologically or genetically has been shown to cause abnormal tubulin polymerization or impaired cell plate formation in Arabidopsis and wheat root tip cells, resulting in defective cytokinesis (Livanos et al., 2012). Similarly, interference with NADPH oxidases, the primary ROS-generating enzymes, and mitogen-activated protein kinases (MAPKs), key mediators of ROS signaling, leads to cytoskeletal disorganization, emphasizing the critical requirement of redox balance during cell division (Mhamdi and Van Breusegem, 2018). ROS also play a pivotal role in regulating cell expansion through modulation of the cell wall. H_2_O_2_, •OH, and O_2_•^−^ present in the apoplast affect cell wall stiffness and extensibility, thereby determining cell expansion rates. Among apoplastic enzymes, NADPH oxidases, amine oxidases, oxalate oxidases, and class III peroxidases contribute to ROS production, with the latter exerting contrasting effects peroxidative activity promotes wall stiffening, whereas hydroxylating activity facilitates wall loosening (Schmidt et al., 2016).

Programmed cell death (PCD) is another vital ROS-mediated process integral to plant development and stress adaptation. PCD ensures the selective elimination of unnecessary or damaged cells during both vegetative and reproductive development, maintaining cellular homeostasis and enhancing plant resilience (Van Aken and Van Breusegem, 2015; Van Durme and Nowack, 2016). It contributes to diverse developmental processes including embryogenesis, tracheary element differentiation, root aerenchyma formation, tapetum degeneration, pollen self-incompatibility, floral organ abscission, leaf senescence, and organ remodeling (Gechev et al., 2006; Singh et al., 2015). Hydrogen peroxide serves as a central signaling molecule orchestrating developmental PCD (dPCD) in various plant tissues, such as ovules, sepals, and petals after fulfilling their physiological roles (Singh et al., 2016). For instance, during daylily petal senescence, increased ROS production driven by enhanced SOD activity and reduced CAT activity triggers dPCD (Singh et al., 2016). In Arabidopsis thaliana, NADPH oxidase RBOHE-generated H_2_O_2_ regulates the timing of tapetal dPCD, which is critical for successful pollen maturation and release (Xie et al., 2014; Van Hautegem et al., 2015). ROS signaling also underpins pollen self-incompatibility (SI) responses in Papaver, where H_2_O_2_ and nitric oxide (NO) accumulation triggers PCD in incompatible pollen, thus preventing self-fertilization. Scavenging these reactive species suppresses SI-induced PCD, underscoring their essential role in the associated signaling cascade that involves ROS, _3_, NO, MAPK activation, and protein phosphorylation (Serrano et al., 2015; Singh et al., 2016). During vegetative development, ROS contribute to the formation of xylem tracheary elements by promoting lignification and secondary wall differentiation. H_2_O_2_ facilitates cross-linking of cell wall polymers and acts as a signaling molecule for secondary wall thickening (Singh et al., 2016). In *Zinnia elegans*, a transient H_2_O_2_ burst during lignification triggers tracheary element differentiation, whereas non-lignifying parenchyma cells supply H_2_O_2_ required for monolignol polymerization. ROS accumulation also serves as an age-dependent trigger for leaf senescence (Khanna-Chopra, 2012). Lipid peroxidation, often induced by lipoxygenases and ROS, intensifies with progressing senescence, accompanied by elevated H_2_O_2_ levels and protein oxidation (Bhattacharjee, 2014). H_2_O_2_ further regulates leaf abscission by inducing cellulase gene expression that facilitates cell wall degradation in the abscission zone. Inhibition of H_2_O_2_ production suppresses cellulase activity and abscission, whereas exogenous H_2_O_2_ enhances both processes, confirming its regulatory role in senescence-associated signaling (Singh et al., 2016).

## 7 ROS signaling

ROS act as crucial signaling molecules that regulate gene expression through complex perception and transduction mechanisms. ROS sensors or receptors initiate signaling cascades that lead to transcriptional reprogramming either via direct activation of receptor proteins, intermediate signaling components, or nuclear transcription factors (Qi et al., 2017). Given the transient and highly reactive nature of ROS, their sensing must occur with exceptional spatial and temporal precision. ROS detection may occur at the apoplastic–plasma membrane interface such as through NADPH oxidase (RBOH) activation which subsequently triggers intracellular signaling cascades that alter gene expression. Alternatively, ROS may directly activate signaling molecules within various subcellular compartments, including chloroplasts, mitochondria, and peroxisomes, thereby influencing nuclear gene expression. While ROS can function within a relatively “linear” signal transduction pathway, they frequently modulate other signaling events at multiple regulatory nodes, suggesting an intricate network of pathway convergence and cross-regulation. Beyond discrete signaling roles, ROS influence broader aspects of cellular redox homeostasis, especially when their concentration rises beyond threshold levels. For instance, organelle ROS such as 1O_2_ generated in plastids serve as retrograde signals that communicate with the nucleus, modulating the expression of stress-responsive and protective genes. Such plastid-to-nucleus signaling exemplifies the pivotal role of ROS in maintaining intracellular communication and coordination between organelles. Substantial inter-organelle cross talk has been reported under stress conditions. During high-light stress, mitochondria facilitate the re-oxidation of excess reducing equivalents produced in plastids, thereby preventing over-reduction of the plastidic electron transport chain and limiting ROS overproduction. Mitochondrial enzymes also support photo respiratory metabolism by salvaging glycolate produced in chloroplasts, thus maintaining redox equilibrium across organelles (Padmasree et al., 2002). Collectively, these findings underscore that ROS are not merely damaging by-products but serve as central messengers integrating metabolic, redox, and gene regulatory networks across cellular compartments.

Plant cells detect ROS through multiple mechanisms. These include unidentified receptor proteins that may directly sense ROS, redox-sensitive molecules like transcription factors such as NPR1 and HSFs, and the direct inhibition of phosphatases by ROS. Each of these pathways contributes to the cell’s ability to respond to oxidative signals effectively. ROS are detected through largely unknown mechanisms or receptors. Reactive species like H_2_O_2_, OH^·^, and O_2_^·–^ interact with cellular targets to initiate signaling. Different ROS types may have distinct sensing mechanisms, given their varied responses and production sites. Detection likely involves redox changes in signaling components, with signals either integrating or remaining specific to each ROS type. ROS sensing triggers downstream signaling events involving _3_ and _3_-binding proteins like calmodulin, along with the activation of G-proteins and phospholipid signaling pathways (Knight and Knight, 2001). The serine/threonine protein kinase OXI1 (Oxidative Signal Inducible 1) plays a pivotal role by activating mitogen-activated protein kinases (MAPKs) 3 and 6 via _3_ (Hirt et al., 2011; Howden et al., 2011). OXI1 expression is upregulated in response to various H_2_O_2_-generating stimuli, and its kinase activity is also enhanced by H_2_O_2_*in vivo* (Petersen et al., 2009). Additionally, OXI1 is essential for plant immunity against *Pseudomonas syringae* in *Arabidopsis*. A MAPK cascade involving MAPK3 and MAPK6 operates downstream of OXI1 to regulate various defense responses to ROS-induced stress (Howden et al., 2011). Additionally, MAPKs are activated by PDK1 through the phospholipase-C/D-phosphatidic-acid pathway. H_2_O_2_ plays a key role in activating several MAPKs, including MPK3 and MPK6 in *Arabidopsis*, via the MAPKKK ANP1 (). Overexpression of ANP1 in transgenic plants has been shown to enhance tolerance to heat shock, freezing, and salt stress. Furthermore, H_2_O_2_ increases the expression of nucleotide diphosphate (NDP) kinase 2 in *Arabidopsis*.

ROS profoundly influence transcriptional reprogramming by modulating the activity and expression of several transcription factor (TF) families, including WRKY, Zat, RAV, GRAS, and MYB (Mittler et al., 2022). These TFs act as central regulators in orchestrating plant responses to oxidative stress and in fine-tuning defense and developmental processes. In microbial systems, redox-sensitive transcriptional regulators such as OxyR in Escherichia coli and Yap1 in yeast represent classical examples of ROS-responsive control mechanisms. These proteins detect oxidative stress through specific cysteine residues that undergo reversible oxidation, leading to conformational changes and activation of antioxidant gene expression. Similarly, in plants, different ROS species selectively oxidize distinct cysteine residues within transcription factors, thereby enabling specific gene expression programs to be activated by the same TF depending on the prevailing oxidative signal (Peng et al., 2021). Microarray analyses of Arabidopsis thaliana exposed to H_2_O_2_ have revealed the presence of H_2_O_2_-responsive cis-regulatory elements within promoters of oxidative stress–induced genes, underscoring the existence of a redox-sensitive transcriptional network (Mhamdi and Van Breusegem, 2018). Functional studies using knockout mutants have demonstrated that the zinc-finger protein Zat12 plays a pivotal role in the expression of ascorbate peroxidase 1 (Apx1) and in mediating tolerance to oxidative stress. Likewise, the zinc-finger paralogs LSD1 (LESION SIMULATING DISEASE 1) and LOL1 (LSD1-LIKE 1) exert antagonistic effects on superoxide dismutase (SOD) activity and O_2_•^−^ accumulation, highlighting their role as positive and negative regulators of ROS signaling (Singh et al., 2016). Another key redox-regulated transcriptional coactivator is NONEXPRESSOR OF PATHOGENESIS-RELATED GENES 1 (NPR1), which serves as a master regulator of salicylic acid (SA)-dependent defense gene expression (Castro et al., 2021; Peng et al., 2021). Under non-stress conditions, NPR1 exists as a cytosolic oligomer stabilized by disulfide bonds involving Cys82 and Cys216. During pathogen attack or redox perturbation, increased cellular reduction potential mediated by thioredoxins and antioxidants converts NPR1 into its monomeric form, allowing it to translocate into the nucleus. Once inside the nucleus, NPR1 interacts with TGA/OBF family bZIP transcription factors, activating the transcription of pathogenesis-related (PR) genes and initiating systemic acquired resistance (SAR). ROS levels tightly regulate this oligomer–monomer equilibrium of NPR1. Elevated ROS maintain NPR1 in its inactive oligomeric state, whereas reductive conditions favor its activation. Importantly, NO acts synergistically with ROS as a redox regulator of NPR1 function, modulating S-nitrosylation and disulfide bond exchange to fine-tune immune signaling (Mittler et al., 2022). Collectively, these findings illustrate how ROS-mediated redox modifications dynamically control transcriptional regulators, enabling plants to perceive oxidative cues and mount appropriate defense and developmental responses.

**Figure 3:**
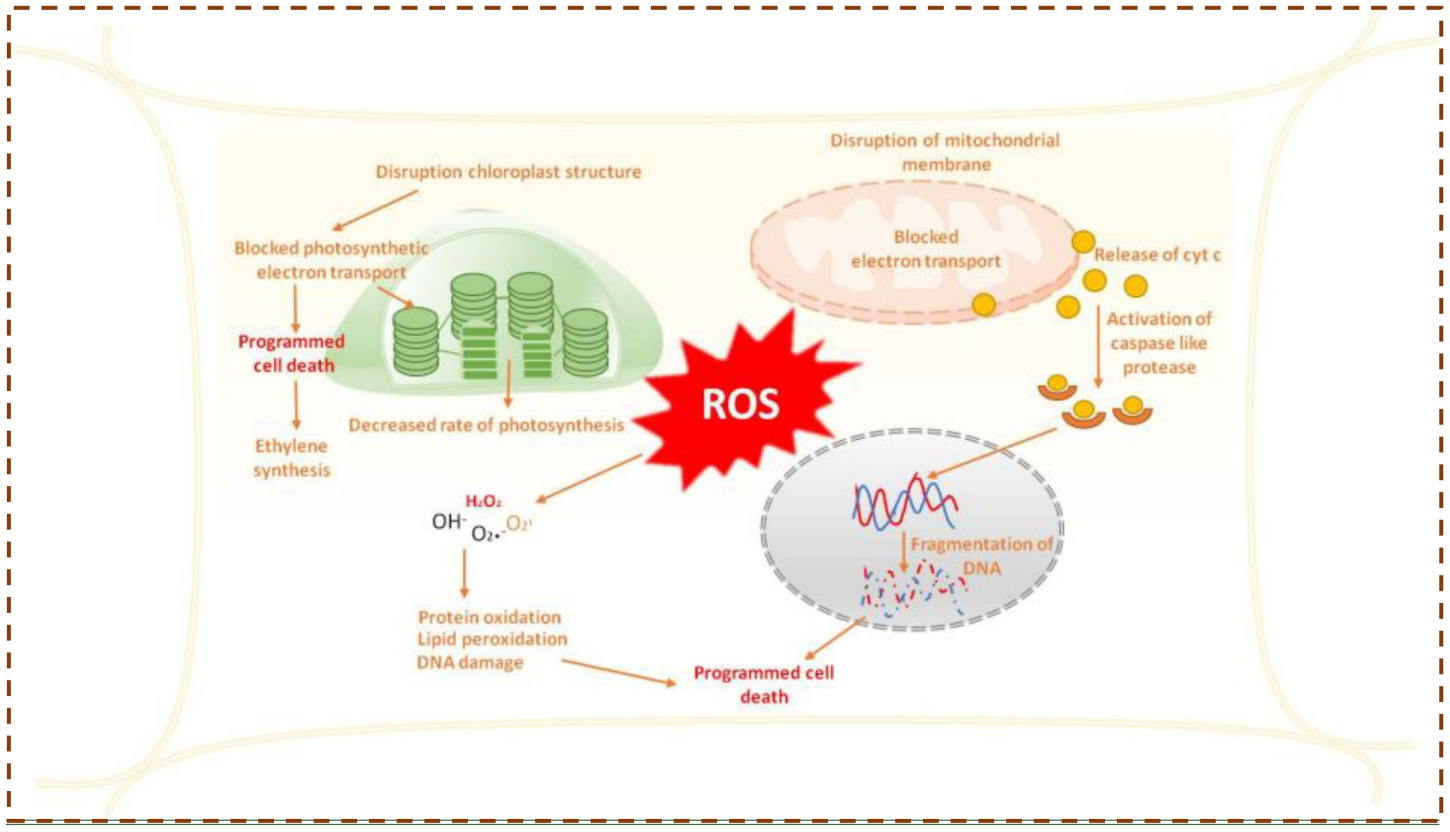
ROS signaling in plants.

Recent studies reveal that various cellular mechanisms produce ROS, whereas the antioxidant system is complex and multifunctional. Research shows that defense hormone signaling in response to catalase deficiency depends on H_2_O_2_-induced changes in glutathione status. Shifting H_2_O_2_ metabolism from catalase to reductive pathways activates DHARs in the cytosol, and knocking out these enzymes weakens cell death and defense responses. Additionally, monodehydroascorbate reductase (MDHAR) may act as a pro-oxidant in certain conditions, and catalase, although crucial for H_2_O_2_ processing, has been linked to promoting autophagy-dependent cell death (Johnston et al., 2015). The effectiveness and redundancy of antioxidant enzymes are essential for system robustness and enable acclimation through various redox signaling pathways (**Figure 3**). Certain, signaling pathways may depend on ROS metabolism, where altered flux and/or changes in antioxidant status act as the perceived signal.

The ability of a cell to switch between ROS-processing pathways helps prevent excessive ROS build-up, meaning that localized stress-induced changes in these molecules might not be easily detectable at the tissue level (Sies, 2017). Some plant studies suggest that H_2_O_2_ concentrations can reach mill molar levels in certain compartments (Sies, 2017). While plant cells may tolerate higher levels than animal cells, peroxisomal H_2_O_2_ concentrations are unlikely to exceed 10 μM even under high production rates (Foyer and Noctor, 2016). ROS signaling regulates both stress acclimation and growth. For instance, catalase-deficient plants show morphological changes influenced by phytohormones like auxins. The distribution of superoxide and H_2_O_2_, controlled by peroxidases, is crucial for root development. This emphasizes the role of ROS in driving plant plasticity in response to environmental stress.

ROS and redox signaling play pivotal roles in regulating both local and systemic defense responses in plants. Through the generation and perception of ROS waves, plants can integrate external stress cues and initiate adaptive signaling across distant tissues, a process known as systemic acquired resistance (SAR) under biotic stress and systemic acquired acclimation (SAA) under abiotic stress. While SAR is typically induced by pathogen infection, SAA is triggered by abiotic challenges such as excess light, heat, or salinity. Despite the differences in their stimuli, both pathways share common mechanistic features, most notably the production of ROS via respiratory burst oxidase homologs (RBOHs) and subsequent activation of redox-sensitive signaling cascades. In *Arabidopsis thaliana*, 10 RBOH isoforms have been identified, among which AtRBOHD and AtRBOHF are the most extensively characterized (Chen and Yang, 2020). These membrane-bound NADPH oxidases are central to ROS-mediated signal amplification and transduction. They participate in diverse physiological and defense-related processes, including ABA-induced stomatal closure, pathogen-triggered defense activation, and systemic signaling during abiotic stresses such as drought, salinity, and mechanical wounding (Chowdhary and Songachan, 2025). The spatiotemporal control of RBOH activity enables plants to produce precisely localized ROS bursts that act as secondary messengers in intercellular communication. A crucial aspect of systemic signaling involves the tight interplay between _3_ and ROS dynamics, forming a self-propagating feedback loop for long-distance signal transmission (Ravi et al., 2023). In this process, _3_ influx activates RBOH-dependent ROS production, whereas ROS, in turn, stimulate _3_ channels in adjacent cells, propagating the signal in a wave-like manner across tissues. This ROS–_3_ wave facilitates rapid systemic communication, enabling distal organs to pre-activate defense genes and stress-responsive pathways even before direct stress exposure. Furthermore, apoplastic redox modifications contribute to systemic signaling and acclimation. Oxidative events in the cell wall and apoplast aid in transmitting light signals from sun-exposed leaves to shaded tissues, thereby enhancing photosynthetic performance and energy balance under fluctuating light conditions. Alterations in cell wall redox homeostasis are also implicated in high-light acclimation and photoinhibition recovery, although the identity of specific redox-sensitive wall components remains to be elucidated. Collectively, these findings underscore that ROS and redox processes not merely are by-products of stress metabolism but act as integral signaling entities that orchestrate whole-plant defense priming and environmental adaptation.

## 8 The hazardous effect of ROS

Cells naturally generate ROS as part of normal physiological processes; however, excessive ROS production can disrupt cellular balance and lead to oxidative stress. This stress arises when the generation of reactive species outweighs the capacity of antioxidant defense mechanisms, resulting in damage to vital biomolecules such as lipids, proteins, and DNA and contributing to the development of various damage to organism.

### 8.1 DNA

Nuclear and mitochondrial DNA possess distinct evolutionary origins, with mitochondrial DNA (mtDNA) tracing back to bacterial genomes engulfed by ancestral eukaryotic cells (Juan et al., 2021). In contemporary eukaryotes, the majority of mitochondrial proteins are encoded by nuclear DNA. Despite being packaged with protective proteins analogous to nuclear chromatin, mtDNA remains particularly susceptible to ROS generated by the adjacent respiratory chain (Nissanka and Moraes, 2018). DNA damage represents a spectrum of physicochemical alterations that compromise the accurate interpretation and transmission of genetic information (Theurey and Pizzo, 2018). Both endogenous and exogenous stresses can induce diverse molecular modifications (Sharma and Sampath, 2019; Auboeuf, 2020). Several studies have emphasized the critical role of ROS originating from both intracellular and environmental sources in DNA damage, identifying it as a central mechanism in carcinogenesis (Srinivas et al., 2019). ROS attack nitrogenous bases and deoxyribose residues within DNA, initiating oxidative reactions that may culminate in mutations, cancer development, apoptosis, necrosis, or hereditary disorders (Juan et al., 2021). DNA fragmentation often results from nucleosome rupture, which disrupts chromatin compaction and coiling (Juan et al., 2021). Because chromatin structure governs gene transcription, any perturbation in its functional integrity can promote mutagenesis. Oxidative damage to DNA induces base alterations, double-strand breaks, and other mutagenic lesions. Hydroxyl radicals directly damage DNA through strand breaks and oxidative modifications of purine and pyrimidine bases (Poetsch, 2020). The process initiates with radical-induced proton abstraction from deoxyribose, leading to multiple reaction intermediates and ribose fragments through hydrogen atom transfer (HAT) (Juan et al., 2021). Oxidation of deoxyribose at the C-4 position proceeds through several intermediate steps, producing a carbon-cantered radical stabilized by resonance with the ring oxygen. The subsequent addition of O_2_ forms a sugar peroxyl radical that converts into a hydroperoxide intermediate. This undergoes a transposition reaction coupled with ring expansion, ultimately decomposing into several breakdown products, including enamine propenal derivatives (Juan et al., 2021). Oxidative lesions in DNA bases arise from abstraction or addition reactions involving free radicals, leading to carbon-centered radicals (Juan et al., 2021).

The hydroxyl radical also induces base-specific damage. In thymine, •OH abstracts a methyl hydrogen at the 5-position, generating a resonance-stabilized carbon radical that, upon oxygen addition and reduction, yields a hydroxymethylene derivative (Juan et al., 2021). Similarly, hydroxyl radicals add to the C-8 position of guanine, producing a radical at N-7 (Juan et al., 2021). This intermediate may undergo reduction followed by ring-opening fragmentation or oxidation to form 8-hydroxyguanine one of the most extensively characterized DNA lesions induced by hydroxyl radicals (Juan et al., 2021). Beyond base oxidation, ROS attack the DNA sugar–phosphate backbone, producing single- and double-strand breaks that are both mutagenic and clastogenic. Such lesions activate the DNA damage response (DDR), a coordinated signaling cascade encompassing damage recognition, checkpoint activation, cell cycle arrest, and subsequent DNA repair, apoptosis, or immune-mediated clearance. The specific type of DNA lesion dictates the pathway engaged for damage recognition and repair (Huang and Zhou, 2020). Mitochondrial diseases arising from mitochondrial dysfunction are particularly severe due to the organelle’s central role in cellular energy metabolism (Zheng et al., 2018). Mutations in mtDNA disrupt the balance between ROS generation and antioxidant defense systems involving SOD, CAT, and glutathione peroxidase. These mutations exacerbate ROS accumulation, leading to oxidative damage within the mitochondrial matrix, which further impairs antioxidant enzymes and diminishes the activity of key protective proteins such as SOD2 (Wang et al., 2018; Zheng et al., 2018).

### 8.2 Lipids

Cell membranes are extremely sensitive to damage caused by ROS due to the presence of polyunsaturated fatty acids. One significant impact of ROS is lipid peroxidation, a process triggered when membrane phospholipids encounter an ROS oxidizing agent (Yadav et al., 2019). During this reaction, the free radical oxidizes an unsaturated lipid chain, resulting in the formation of a hydroperoxidized lipid and an alkyl radical (Yadav et al., 2019). This lipid peroxidation leads to structural changes in the membrane, altering its fluidity and compromising its integrity. The process begins with a hydroxyl radical attacking specific bis-allylic positions in the fatty acid side chains, generating an alkyl radical (Yadav et al., 2019).

Lipid peroxidation begins with the initiation phase, where a free radical attacks a carbon in the aliphatic chain of a polyunsaturated fatty acid (PUFA). This leads to hydrogen abstraction from the methylene group (–CH_4_–) positioned between double bonds, generating a reactive radical species (Kao et al., 2020). The radical is stabilized through resonance with the double bonds. In the propagation phase, a chain reaction unfolds, amplifying oxidative damage. The initial radical reacts with oxygen to form a peroxyl radical (LOO•), which further interacts with adjacent PUFAs, producing hydroperoxides and additional alkyl radicals (Kao et al., 2020). This continuous cycle escalates lipid degradation, affecting an increasing number of fatty acids. Lipid peroxidation generates toxic byproducts like hydroxynonenal. During this process, peroxyl radicals can undergo cyclization, forming four-membered cyclic peroxides. The extent of peroxidation increases with the degree of unsaturation in polyunsaturated fatty acids, making highly unsaturated lipids more susceptible to oxidative damage (Juan et al., 2021).

During lipid peroxidation, toxic aldehydes like malondialdehyde (MDA) and hydroxynonenal (HNE) are produced. These reactive compounds interact with amino groups (–NH_2_) in proteins and DNA bases, leading to the formation of adducts that can cause mutations (Yang et al., 2019). MDA, in particular, can form covalent bonds with lysine residues on two different proteins or within the same protein, resulting in imine derivatives or Schiff bases after water elimination. Additionally, MDA can react with DNA bases, especially guanine, forming mutagenic lesions (Juan et al., 2021). This reaction proceeds via dehydration, resulting in the formation of an enamine derivative that eventually cyclizes into a six-membered ring. In the case of hydroxynonenal, a 1,4-addition reaction occurs between the α,β-unsaturated aldehyde region and the amino group of guanine, followed by imine formation and subsequent cyclization (Juan et al., 2021).

### 8.3 Proteins and enzymes

Proteins and enzymes are intricate, large molecules essential for various biological functions. Encoded by both nuclear and mitochondrial DNA, they play a crucial role in maintaining cellular activities, as well as supporting the structure, function, and regulation of tissues and organs (Juan et al., 2021). One significant consequence of oxidative stress is the structural damage it inflicts on proteins, leading to the loss of enzymatic activity and disruptions in metabolic regulation (Juan et al., 2021). Over the past two decades, research on the link between lipid peroxidation and neurodegenerative diseases has expanded significantly, especially with advancements in proteomics (Milkovic et al., 2019). This field has provided deeper insights into disease mechanisms, not only in neurodegeneration but also in tumor biology, highlighting the impact of protein modifications. Unlike nucleic acids, oxidized proteins cannot be repaired and must be degraded or processed by the proteasome to prevent their accumulation and potential interference with cellular functions. ROS can lead to the oxidation of amino acid residues, cleavage of peptide bonds, and aggregation of proteins (Milkovic et al., 2019).

Protein oxidation begins when the hydroxyl radical abstracts a hydrogen atom from the protein, generating an alkyl radical stabilized by resonance with the carboxyl group. This alkyl radical then reacts with oxygen, leading to the formation of peroxide radical (Juan et al., 2021). The peroxide radical further abstracts hydrogen from a nearby protein, resulting in the formation of a hydroperoxide and another alkyl radical. In the presence of ferrous iron, the hydroperoxide is reduced to an alkoxy radical, which subsequently abstracts hydrogen from an adjacent protein, producing hydroxy amino acid derivatives (Milkovic et al., 2019. The cleavage of the alkoxy radical generates various protein carboxy radicals and alkyl radicals. Under low oxygen conditions, these alkyl radicals promote the formation of protein aggregates (Juan et al., 2021).

## 9 ROS cross talk between hormones

### 9.1 Strigolactones

Strigolactones (SLs) were a unique class of terpenoid lactones derived from carotenoids, exhibiting remarkable protective capabilities. As a class of plant hormones, SLs showed great potential as key biomolecules in mitigating oxidative stress in plants (Mansoor et al., 2024). They contributed to reducing oxidative stress by promoting the production and accumulation of antioxidants, which effectively scavenged ROS and counteracted their damaging effects (Wani et al., 2023). SLs played a crucial role in detoxifying ROS whereas regulating the expression of genes associated with antioxidant defense pathways, maintaining a balance between ROS production and elimination (Wani et al., 2023). SLs demonstrated efficacy in ameliorating the detrimental effects of low light stress by enhancing the activity of antioxidant enzymes and maintaining the efficiency of photosynthesis, both integral components of the oxidative defense system in plants (Mansoor et al., 2024). SLs also played a crucial role in protecting plants from drought stress. They effectively reduced ROS levels, improved water retention, and strengthened cell membranes in response to drought conditions. Similarly, under heat or cold stress, SLs helped mitigate oxidative damage by triggering ROS-scavenging mechanisms and reinforcing the overall oxidative defense system of plants (Min et al., 2019; Sedaghat et al., 2020). The application of exogenous SLs preserved chlorophyll content and sustained the photosynthetic rate in apple seedlings under KCl stress. It enhanced the activity of POX and CAT enzymes, which effectively decreased ROS, induced by KCl stress, facilitated proline accumulation, and ensured osmotic balance (Zheng et al., 2021). Exogenous SL analog GR24 mitigated salt stress in cucumber by reducing ROS content by improving ascorbate-glutathione (AsA-GSH) cycle (Zhang et al., 2022). Water deficit conditions produce oxidative stress by increasing ROS and MDA content. However, SL application increased drought tolerance in *Brassica rapa* by improving the antioxidant defense system that reduced ROS and MDA content (Ali et al., 2023). Nutrient deprivation also promoted the accumulation of toxic ROS (Banerjee and Roychoudhury, 2018). Phosphorous and nitrogen limiting conditions activated NADPH oxidases in *Medicago truncatula* roots, leading to a high expression of SL biosynthetic genes (Bonneau et al., 2013). This condition-specific accumulation of SLs suggested that these molecules might have had the capacity to scavenge ROS or promoted the accumulation of downstream osmolytes and antioxidants to maintain cellular osmolyte content (Banerjee and Roychoudhury, 2018).

### 9.2 Salicylic acid

Salicylic acid (SA) is a powerful phytohormone that contributes to the production of ROS in excess (acting as a pro-oxidant) while also boosting the activity of ROS-scavenging enzymes (antioxidants) during stressful conditions (Arif et al., 2020). Interestingly, the effect of SA on ROS production is time- and concentration-dependent, where it can either promote ROS production (acting as a pro-oxidant) or enhance ROS scavenging (acting as an antioxidant) in plants. The balance between these pro-oxidant and antioxidant activities is essential in determining whether plants survive or succumb to abiotic stresses. Apoplastic ROS have been identified as key regulators of cell death through their interactions with various signaling pathways, including those mediated by SA (Khan et al., 2015). Both internally produced and externally applied SA has been shown to influence antioxidant metabolism and exert strict control over cellular ROS levels (Khan et al., 2015). SA triggers an increase in ROS production, particularly H_2_O_2_, which serves as a key signal to activate the antioxidant defense system, helping the plants to cope with abiotic stress. SA acts as a key regulator of ROS that plays a crucial role in maintaining equilibrium to prevent excessive ROS accumulation that could lead to oxidative stress (Lukan and Coll, 2022). However, ROS not only function downstream of SA signaling but also are considered central to a self-amplifying loop that governs SA signaling and modulates interactions among various phytohormones (Lukan and Coll, 2022). The nature of this cross talk includes the points where SA influences ROS signaling and vice versa, as well as the overall regulatory outcome, depending on the source of ROS (Lukan and Coll, 2022). In *Brassica napus*, SA reduced ROS content during drought stress by increasing activities of antioxidant and proline content. Moreover, the NPR1-dependent signaling pathway regulated by SA and proline synthesis work together as an integrated mechanism for redox control under abiotic stress (Lee et al., 2019). Upon pathogen attack, tobacco rapidly synthesizes SA, which inhibits catalase, triggering an H_2_O_2_ burst (Liu et al., 2022). This SA-induced H_2_O_2_ acts as a second messenger, activating defense proteins and systemic acquired resistance (SAR) (Liu et al., 2022). Since H_2_O_2_ also plays a role in abiotic stress responses, SA-induced H_2_O_2_ may enhance stress tolerance. For instance, SA pretreatment in rice roots increased H_2_O_2_ levels, boosting antioxidant defenses and reducing oxidative damage from cadmium stress (Liu et al., 2022). SA treatment was crucial in maintaining a stable balance between ROS and the antioxidant defense system, including catalase, peroxidase, and superoxide dismutase (Arif et al., 2023). SA application enhanced antioxidant enzyme activity. This improvement led to reduced ROS and lower MDA accumulation (Nazir et al., 2024). In rice, tomato, and wheat, SA regulated the AsA-GSH cycle and glyoxalase system, enhanced photosynthesis, and strengthened the antioxidant defense system, which alleviated abiotic stress by reducing electrolyte leakage and lowering ROS levels (Delaix et al., 2025).

### 9.3 Brassinosteroids

The generation and detoxification of ROS have been associated with brassinosteroids (BRs), and their interaction plays a crucial role in regulating stress tolerance (Delaix et al., 2025). Higher BR levels boost H_2_O_2_ production, which acts as a signaling molecule, activating stress-related molecules like transcription factors, dehydrins, HSPs, and antioxidant enzymes to reduce ROS under stress conditions (Yaqoob et al., 2022). Recent studies show that BRs regulate root tip stem cell activity through ROS. BR binding to the BRI1 receptor kinase raises H_2_O_2_ levels, which then oxidatively modify BZR1 and BES1, key transcription factors in BR signaling. This modification boosts BZR1 activity by promoting its interaction with PIF4 and ARF6, aiding root meristem development (Lv et al., 2018). Mutants in the BR biosynthetic pathway have revealed a strong interaction between BR signaling and ROS production. In BR mutants, the ratios of ascorbic acid/dehydroascorbic acid (AsA/DHA) and glutathione/glutathione disulfide (GSH/GSSG) are reduced. However, BR application increases the activity of antioxidant enzymes, defense-related genes, and the AsA/DHA and GSH/GSSG ratios (Zhou et al., 2014). In *Glycine max*, exogenous BR application increased the activity of POD and SOD which reduced ROS under low water conditions (Zhang et al., 2011). In rice seedlings, BR application under salt stress led to a significant increase in the activity of SOD, GR, and CAT, with a slight rise in APX activity and decreased ROS content (Yaqoob et al., 2022). BR-induced stress tolerance was closely linked to improvements in CO_2_ assimilation, photoprotection, antioxidant capacity (both enzymatic and non-enzymatic), redox balance, ROS scavenging, defense mechanisms, secondary metabolism, detoxification, and autophagy (Ahammed et al., 2020). In cucumber, BR treatment triggered the expression of regulatory genes like RBOH, MAPK1, and MAPK3, as well as genes associated with defense and antioxidant responses (Xia et al., 2009). In EBL-primed rice plants, the gene expression and enzymatic activities of SOD and POD increased to counter ROS production under Cr stress (Basit et al., 2021). BR was involved in root growth and interacted with ethylene and ROS in the *A. thaliana* det2–9 mutant, which exhibited a short-root phenotype due to impaired BR synthesis. The mutant accumulated higher levels of ethylene and ROS, leading to inhibited root growth. These findings revealed how BRs influenced root development through cross-regulation with ethylene and ROS signaling pathways (Lv et al., 2018). BR-deficient mutants (*d^im* and *bzr1*) exhibited reduced pollen viability, pollen germination, and seed number, whereas overexpression of DWARF (DWF) or BRASSINAZOLE RESISTANT 1 (BZR1) had the opposite effects. Loss or gain of function in DWF or BZR1 disrupted ROS production and PCD timing in tapetal cells, resulting in delayed or premature degeneration (Yan et al., 2020).

### 9.4 Jasmonic acid

The ROS wave orchestrates a wide range of physiological, molecular, and metabolic responses across different plant organs and plays a pivotal role in systemic acquired acclimation (SAA) under stress conditions. Moreover, it exhibits intricate interactions with several phytohormones, including JA, ABA, and SA (Myers et al., 2023). JA acts as a negative regulator of the ROS wave during high light (HL) stress and wounding, whereas SA functions as a positive modulator enhancing ROS wave propagation. Ethylene (ET) influences ROS wave dynamics in response to wounding but not during HL stress, whereas strigolactones (SLs) appear to play no role in either condition. The redox-responsive protein NPR1 is indispensable for systemic but not local ROS accumulation and the subsequent acclimation to HL stress. This observation suggests that ROS wave-induced redox perturbations modulate systemic gene expression through SA or ROS-dependent signaling pathways, with JA acting antagonistically to suppress these responses (Myers et al., 2023). Several stress and hormone-responsive genes have been identified in this context, including those encoding 12-oxophytodienoate reductase (OPR) involved in JA biosynthesis, 1-aminocyclopropane-1-carboxylic acid oxidase (ACO) participating in ET biosynthesis, and peroxidases responsible for ROS generation. Exogenous JA application has been shown to suppress aphid reproduction in cucumber leaves while significantly upregulating OPR11 expression (Qi et al., 2020). Furthermore, COI1-mediated JA signaling plays a central role in fine-tuning both enzymatic and non-enzymatic antioxidant defense components, in addition to modulating pathogen-associated molecular pattern (PAMP)-triggered immunity in plants (Kadam and Barvkar, 2024).

### 9.5 Karrikins

Karrikins (KARs) are a group of closely related chemical compounds found in charred or burnt plant material and smoke. They can also be produced through the pyrolysis of cellulose and simple sugars. Six karrikin molecules have been identified and designated as KAR^1^, KAR^2^, KAR^3^, KAR^4^, KAR^5^, and KAR^6^, with KAR^1^ to KAR^4^ recognized as the most biologically active forms (Shah et al., 2020). Karrikins are known to promote seed germination and photomorphogenesis, while inhibiting hypocotyl elongation in various plant species (Shah et al., 2020). Due to their structural similarity with SLs, KARs are believed to play a role in enhancing abiotic stress tolerance in plants. Emerging evidence suggests that KARs contribute to maintaining ROS homeostasis by modulating antioxidant enzyme activities and reducing oxidative damage under stress conditions. This ROS-regulating ability positions KARs as key signaling molecules in plant stress responses (Banerjee and Roychoudhury, 2018). Treatment with KAR1 significantly enhanced the levels of all antioxidants in *Sapium sebiferum* seedlings exposed to salt and osmotic stress, indicating that karrikins help alleviate abiotic stress by boosting the antioxidant defense system. Therefore, karrikins play a protective role under stress conditions by modulating endogenous H_2_O_2_ levels, reducing electrolyte leakage, and maintaining membrane integrity (Shah et al., 2020). KAR1 and GA_3_ were found to elevate ascorbate and dehydroascorbate levels while decreasing glutathione and oxidized glutathione. They also enhanced the activity of ascorbate peroxidase and glutathione reductase. These compounds regulated ROS–antioxidant balance in both embryos and aleurone layers (Kępczyński, 2018). KAR mitigated ROS produced by cold stress via activating enzymatic and non-enzymatic antioxidant defense machinery in *Arabidopsis* (Shah et al., 2020). Black cumin seedlings pretreated with KAR effectively countered salinity-induced effects by lowering ROS levels and boosting the activities of enzymes involved in the ascorbate glutathione cycle, along with other antioxidants like CAT, POX, and SOD, as well as osmoprotectants such as proline (Sharifi and Shirani Bidabadi, 2020).

## 10 ROS detection in plants

ROS can be classified according to their photochemical generation mechanisms. Among the most commonly used probes for detecting total ROS is 2′,7′-dichlorodihydrofluorescein (H_2_DCF). In its diacetate form (H_2_DCFDA), the molecule is membrane-permeable, allowing efficient cellular uptake (Arif et al., 2023). Once inside the cell, intracellular esterases deacetylate H_2_DCFDA to non-fluorescent H_2_DCF, which becomes trapped within the cytoplasm. Upon oxidation by ROS, H_2_DCF is converted to 2′,7′-dichlorofluorescein (DCF), a highly fluorescent compound. The rate of DCF formation indicative of intracellular ROS levels can be quantified through fluorometry or fluorescence microscopy (Arif et al., 2023). Consequently, DCFDA-based assays are among the most robust and widely accepted methods for assessing overall ROS accumulation in biological systems (Mahalingam et al., 2006). For histochemical localization of specific ROS species in plant tissues, nitroblue tetrazolium (NBT) and 3,3′-diaminobenzidine (DAB) are frequently employed. NBT reacts selectively with superoxide radicals (O_2_•^−^), resulting in the formation of a blue or purple formazan precipitate that marks superoxide accumulation. Conversely, DAB reacts with H_2_O_2_ to yield a distinctive brown polymerized product, providing a visual marker of H_2_O_2_ localization (Arif et al., 2023). Lipid peroxidation represents one of the most deleterious consequences of ROS overproduction, as it compromises membrane integrity and cellular function (Catalá and Díaz, 2016). This process proceeds through three sequential phases’ initiation, propagation, and termination. During initiation, reactive species such as hydroxyl or peroxyl radicals abstract hydrogen atoms from polyunsaturated fatty acids (PUFAs), generating lipid radicals (Catalá, 2006; Catalá and Díaz, 2016). These radicals rapidly react with molecular oxygen to form lipid peroxyl radicals and lipid hydroperoxides (LOOH), perpetuating a chain reaction that amplifies membrane damage. In the presence of transition metals, LOOH decomposes into alkoxyl radicals, which further propagate lipid oxidation by abstracting additional hydrogen atoms (Jambunathan, 2010). Quantification of lipid peroxidation is commonly performed using the thiobarbituric acid reactive substances (TBARS) assay. This method measures malondialdehyde (MDA), a stable end product of PUFA degradation, which reacts with thiobarbituric acid (TBA) to form a chromogenic adduct detectable by spectrophotometry (Papastergiadis et al., 2012). In plant systems, a modified TBARS assay is routinely employed to evaluate oxidative membrane damage associated with various abiotic and biotic stresses.

These analytical and histochemical techniques have become indispensable tools in plant stress physiology for elucidating redox dynamics under both abiotic and biotic stress conditions. DCFDA-based fluorometric assays enable quantitative assessment of total ROS accumulation in response to drought, salinity, heavy metal toxicity, or nanoparticle exposure, providing insights into oxidative homeostasis at the cellular level. Complementarily, NBT and DAB staining facilitates spatial visualization of superoxide and H_2_O_2_ distribution in leaf and root tissues, thereby revealing tissue-specific redox patterns during stress adaptation. The TBARS assay, through quantification of MDA, serves as a robust biochemical indicator of lipid peroxidation and membrane injury. Collectively, these methods allow researchers to correlate ROS production with physiological, biochemical, and molecular alterations, thereby advancing our understanding of oxidative signaling, antioxidant defense activation, and overall stress tolerance mechanisms in plants.

## 11 Conclusion

ROS are indispensable yet paradoxical components of plant biology, functioning as both damaging oxidants and critical signaling molecules. Their dynamic production and detoxification govern a wide spectrum of physiological and molecular processes, from growth and development to defense and acclimation. ROS waves integrate with hormonal networks particularly JA, SA, ABA, and ET to coordinate local and systemic responses to environmental cues. Moreover, their cross talk with _3_, NO, and MAPK cascades ensures precise modulation of stress signaling and transcriptional reprogramming across plant tissues. Advanced biochemical and histochemical detection techniques such as DCFDA-based fluorometric assays, NBT and DAB staining, and TBARS quantification have become indispensable tools for investigating ROS dynamics under various abiotic and biotic stress conditions. These approaches allow both spatial visualization and quantitative assessment of ROS accumulation, lipid peroxidation, and membrane integrity, providing valuable insights into oxidative homeostasis and defense activation in plants. Overall, this review highlights the multifaceted nature of ROS, encompassing their chemistry, generation sites, and dual functional roles in plant systems. The complex interplay between ROS and phytohormones underscores their pivotal role in maintaining cellular equilibrium and stress adaptability. However, despite substantial progress, the precise molecular mechanisms governing ROS perception, signal transduction, and downstream gene regulation remain only partially understood. Future research should focus on elucidating how plants sense and discriminate ROS signals, and how these are integrated into broader signaling networks. The development of real-time, high-resolution detection technologies, combined with advanced genetic, transcriptomic, and imaging tools, will be vital for decoding spatiotemporal ROS dynamics. Ultimately, harnessing ROS-mediated signaling offers a promising frontier for engineering crop plants with enhanced resilience and productivity under challenging environmental conditions.
